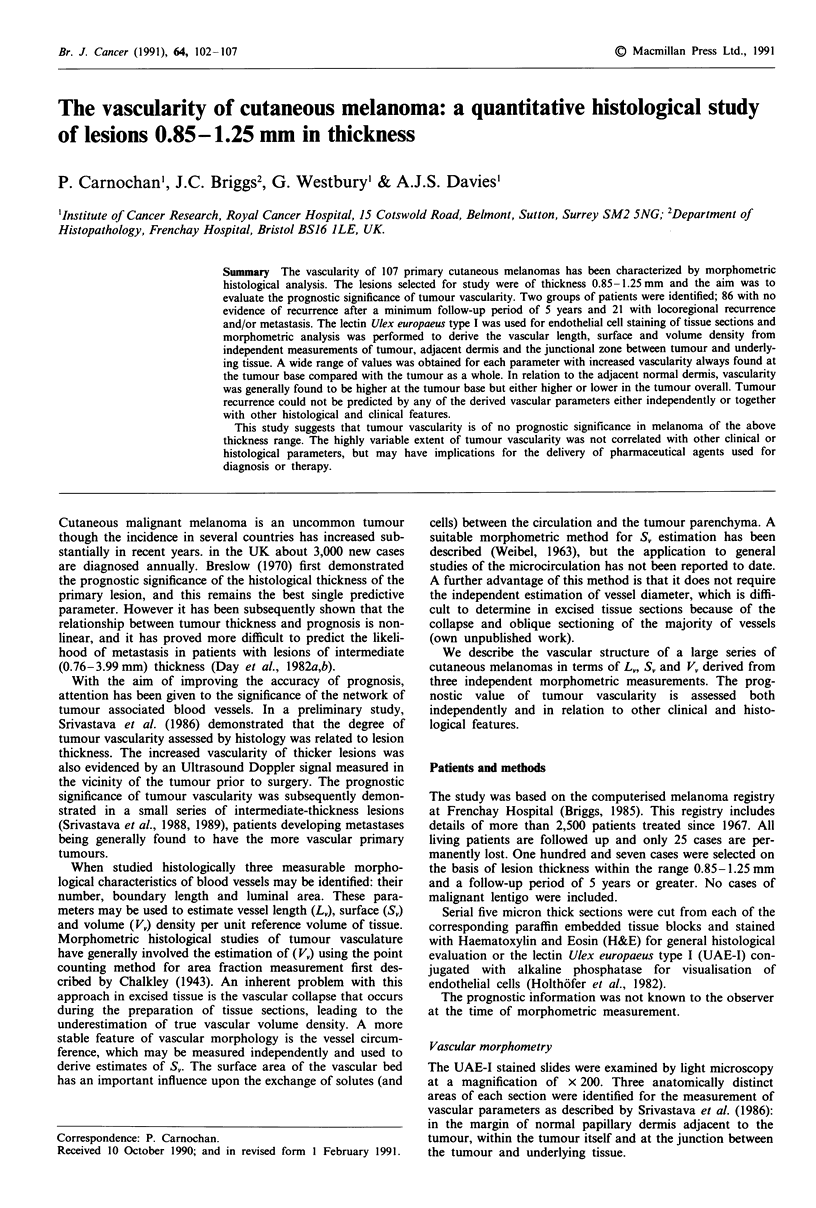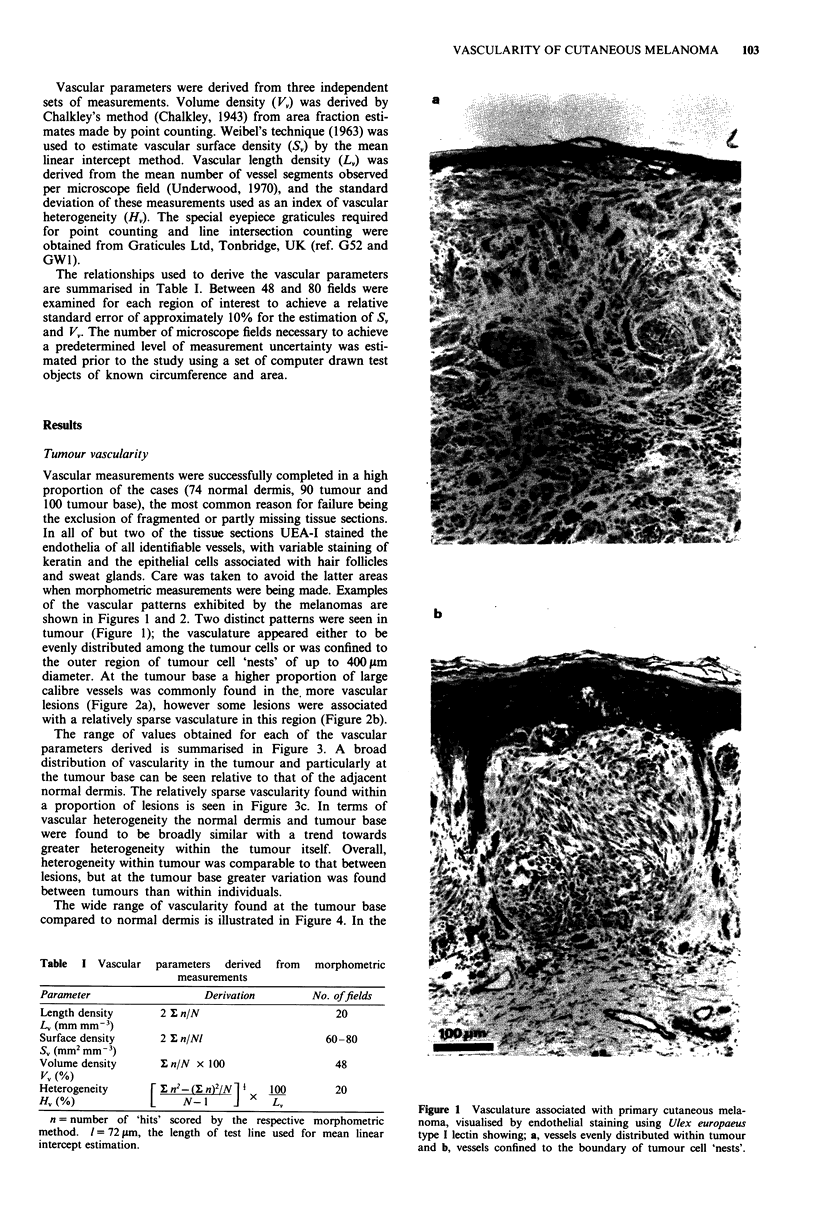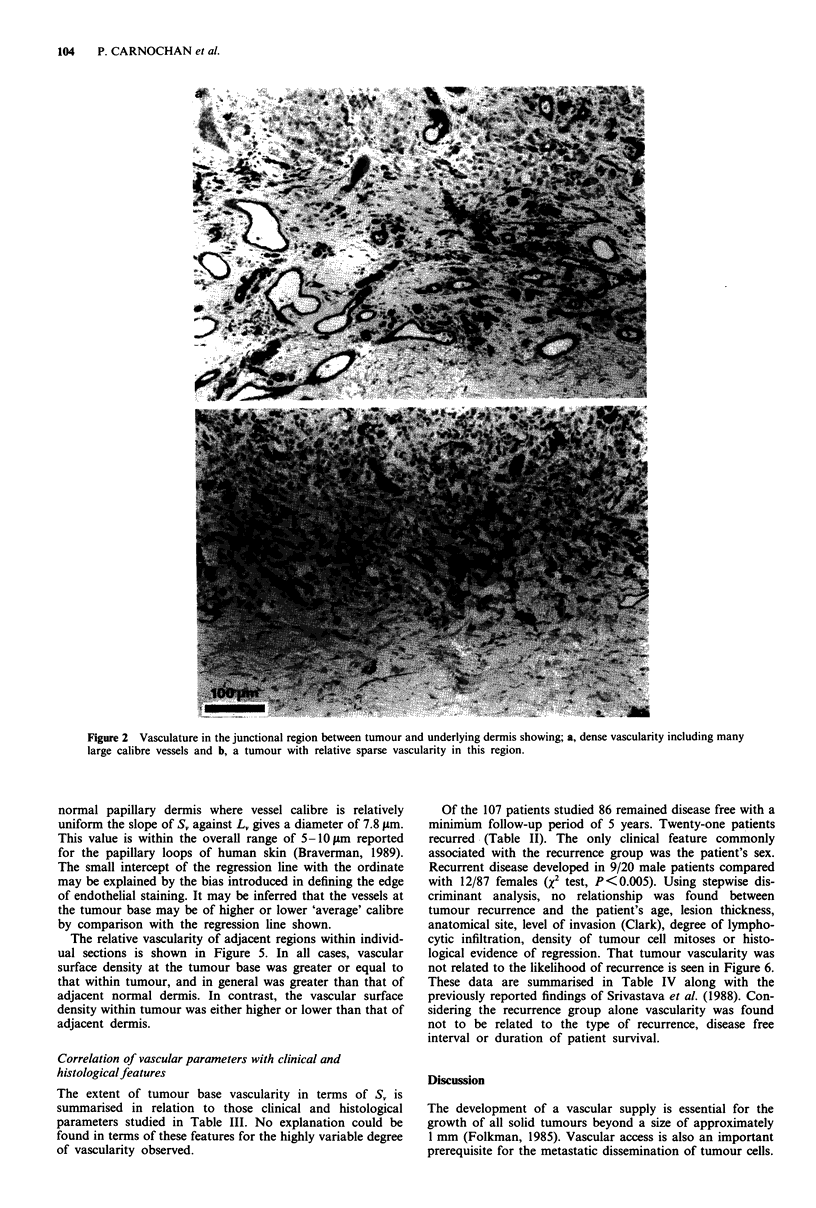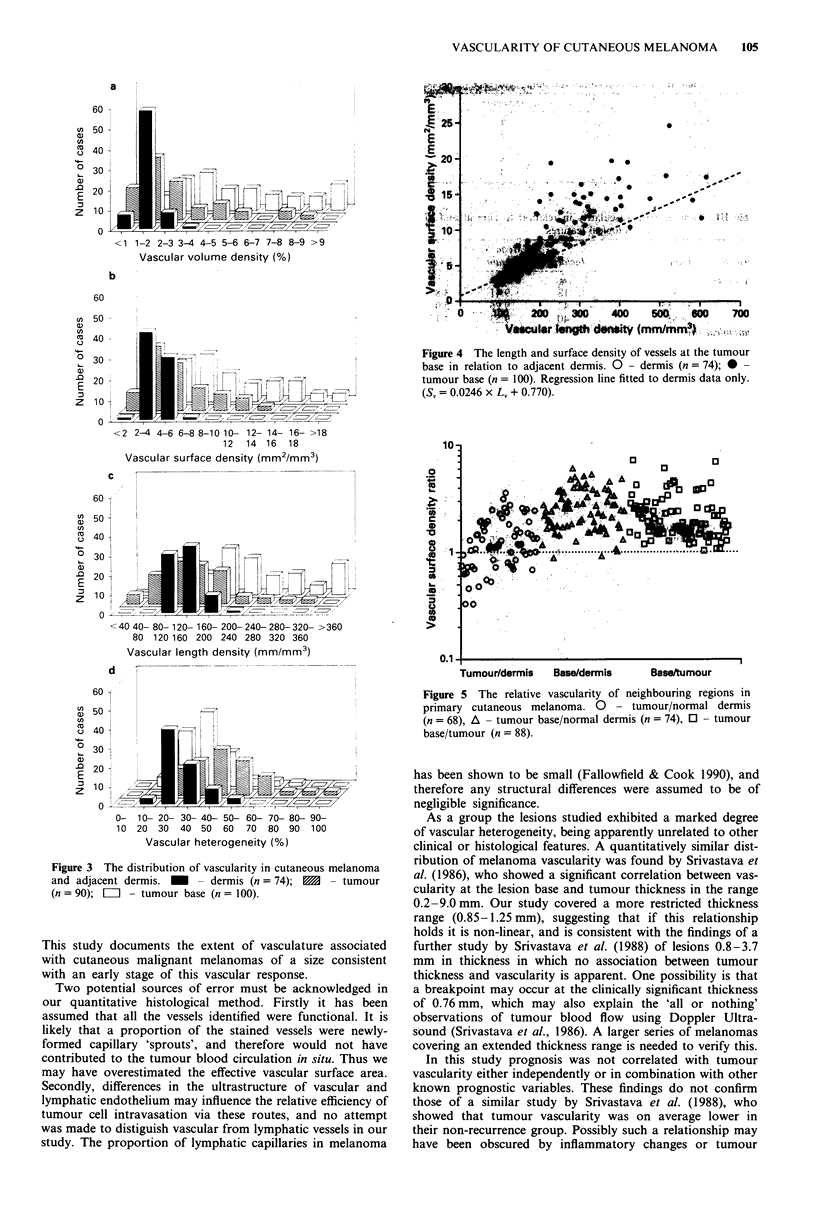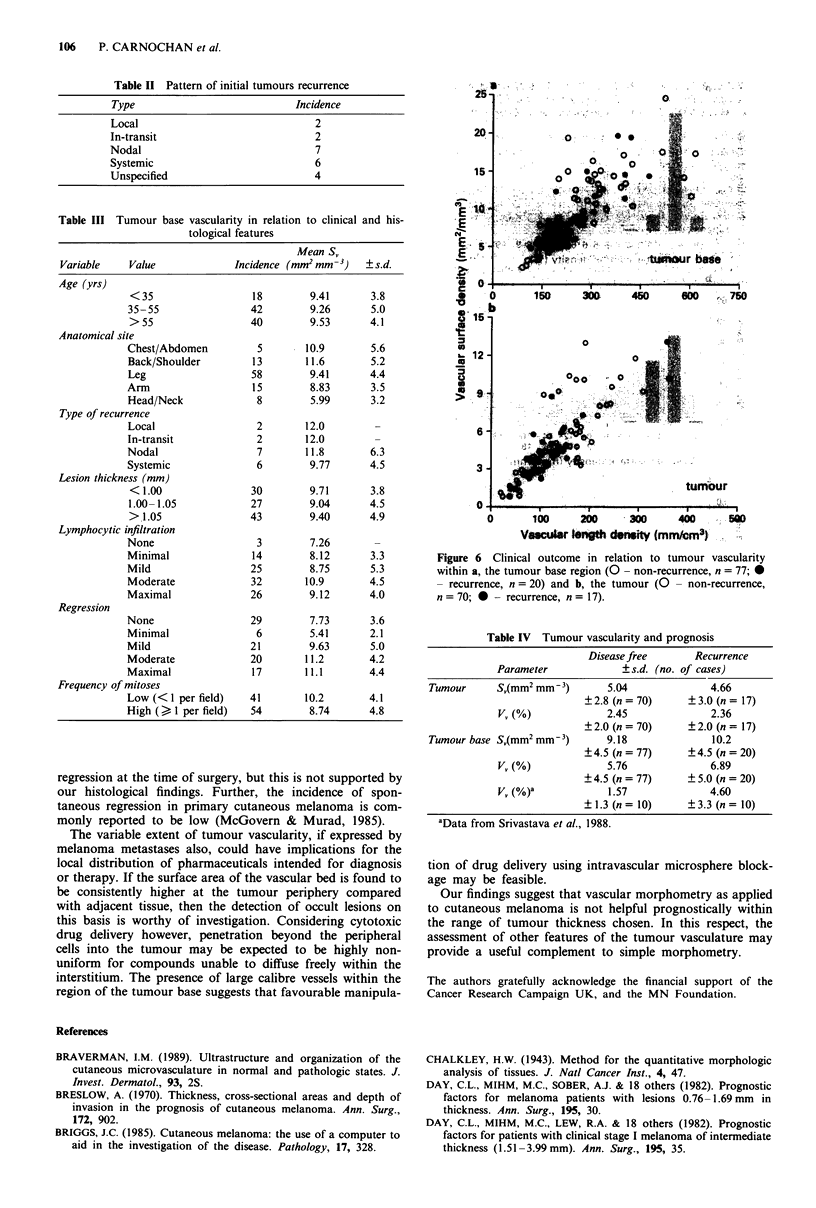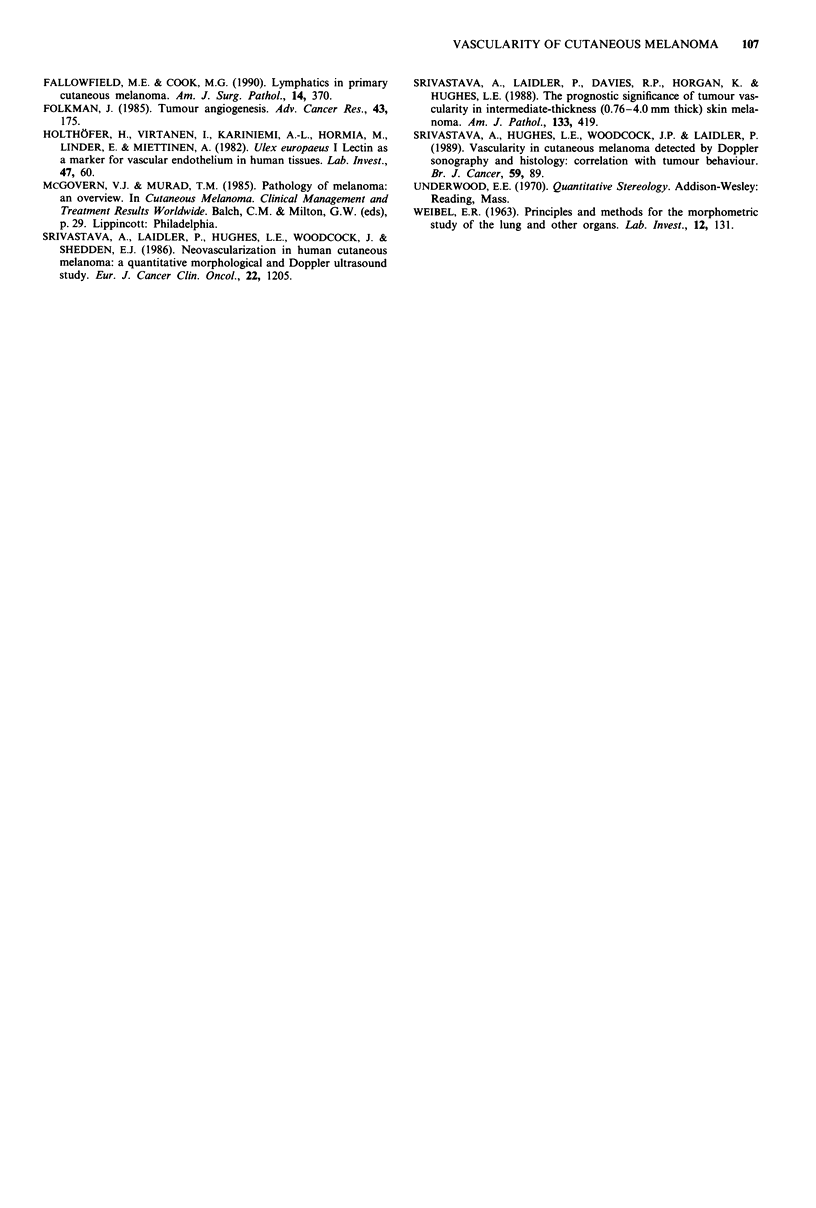# The vascularity of cutaneous melanoma: a quantitative histological study of lesions 0.85-1.25 mm in thickness.

**DOI:** 10.1038/bjc.1991.250

**Published:** 1991-07

**Authors:** P. Carnochan, J. C. Briggs, G. Westbury, A. J. Davies

**Affiliations:** Institute of Cancer Research, Royal Cancer Hospital, Sutton, Surrey, UK.

## Abstract

**Images:**


Br. J. Cancer (1991), 64, 102–107 © Macmillan Press Ltd., 1991

### The vascularity of cutaneous melanoma: a quantitative histological study of lesions 0.85–1.25 mm in thickness

P. Carnochan[1], J.C. Briggs[2], G. Westbury[1] & A.J.S. Davies[1]

[1]*Institute of Cancer Research, Royal Cancer Hospital, 15 Cotswold Road, Belmont, Sutton, Surrey SM2 5NG;* [2]*Department of Histopathology, Frenchay Hospital, Bristol BS16 1LE, UK.*

**Summary** The vascularity of 107 primary cutaneous melanomas has been characterized by morphometric histological analysis. The lesions selected for study were of thickness 0.85–1.25 mm and the aim was to evaluate the prognostic significance of tumour vascularity. Two groups of patients were identified; 86 with no evidence of recurrence after a minimum follow-up period of 5 years and 21 with locoregional recurrence and/or metastasis. The lectin *Ulex europaeus* type I was used for endothelial cell staining of tissue sections and morphometric analysis was performed to derive the vascular length, surface and volume density from independent measurements of tumour, adjacent dermis and the junctional zone between tumour and underlying tissue. A wide range of values was obtained for each parameter with increased vascularity always found at the tumour base compared with the tumour as a whole. In relation to the adjacent normal dermis, vascularity was generally found to be higher at the tumour base but either higher or lower in the tumour overall. Tumour recurrence could not be predicted by any of the derived vascular parameters either independently or together with other histological and clinical features.

This study suggests that tumour vascularity is of no prognostic significance in melanoma of the above thickness range. The highly variable extent of tumour vascularity was not correlated with other clinical or histological parameters, but may have implications for the delivery of pharmaceutical agents used for diagnosis or therapy.

Cutaneous malignant melanoma is an uncommon tumour though the incidence in several countries has increased substantially in recent years. in the UK about 3,000 new cases are diagnosed annually. Breslow (1970) first demonstrated the prognostic significance of the histological thickness of the primary lesion, and this remains the best single predictive parameter. However it has been subsequently shown that the relationship between tumour thickness and prognosis is non-linear, and it has proved more difficult to predict the likelihood of metastasis in patients with lesions of intermediate (0.76–3.99 mm) thickness (Day *et al.*, 1982*a*,*b*).

With the aim of improving the accuracy of prognosis, attention has been given to the significance of the network of tumour associated blood vessels. In a preliminary study, Srivastava *et al.* (1986) demonstrated that the degree of tumour vascularity assessed by histology was related to lesion thickness. The increased vascularity of thicker lesions was also evidenced by an Ultrasound Doppler signal measured in the vicinity of the tumour prior to surgery. The prognostic significance of tumour vascularity was subsequently demonstrated in a small series of intermediate-thickness lesions (Srivastava *et al.*, 1988, 1989), patients developing metastases being generally found to have the more vascular primary tumours.

When studied histologically three measurable morphological characteristics of blood vessels may be identified: their number, boundary length and luminal area. These parameters may be used to estimate vessel length ($L_v$), surface ($S_v$) and volume ($V_v$) density per unit reference volume of tissue. Morphometric histological studies of tumour vasculature have generally involved the estimation of ($V_v$) using the point counting method for area fraction measurement first described by Chalkley (1943). An inherent problem with this approach in excised tissue is the vascular collapse that occurs during the preparation of tissue sections, leading to the underestimation of true vascular volume density. A more stable feature of vascular morphology is the vessel circumference, which may be measured independently and used to derive estimates of $S_v$. The surface area of the vascular bed has an important influence upon the exchange of solutes (and cells) between the circulation and the tumour parenchyma. A suitable morphometric method for $S_v$ estimation has been described (Weibel, 1963), but the application to general studies of the microcirculation has not been reported to date. A further advantage of this method is that it does not require the independent estimation of vessel diameter, which is difficult to determine in excised tissue sections because of the collapse and oblique sectioning of the majority of vessels (own unpublished work).

We describe the vascular structure of a large series of cutaneous melanomas in terms of $L_v$, $S_v$ and $V_v$ derived from three independent morphometric measurements. The prognostic value of tumour vascularity is assessed both independently and in relation to other clinical and histological features.

### Patients and methods

The study was based on the computerised melanoma registry at Frenchay Hospital (Briggs, 1985). This registry includes details of more than 2,500 patients treated since 1967. All living patients are followed up and only 25 cases are permanently lost. One hundred and seven cases were selected on the basis of lesion thickness within the range 0.85–1.25 mm and a follow-up period of 5 years or greater. No cases of malignant lentigo were included.

Serial five micron thick sections were cut from each of the corresponding paraffin embedded tissue blocks and stained with Haematoxylin and Eosin (H&E) for general histological evaluation or the lectin *Ulex europaeus* type I (UAE-I) conjugated with alkaline phosphatase for visualisation of endothelial cells (Holthöfer *et al.*, 1982).

The prognostic information was not known to the observer at the time of morphometric measurement.

### *Vascular morphometry*

The UAE-I stained slides were examined by light microscopy at a magnification of × 200. Three anatomically distinct areas of each section were identified for the measurement of vascular parameters as described by Srivastava *et al.* (1986): in the margin of normal papillary dermis adjacent to the tumour, within the tumour itself and at the junction between the tumour and underlying tissue.

Correspondence: P. Carnochan.
Received 10 October 1990; and in revised form 1 February 1991.

Vascular parameters were derived from three independent sets of measurements. Volume density ($V_v$) was derived by Chalkley's method (Chalkley, 1943) from area fraction estimates made by point counting. Weibel's technique (1963) was used to estimate vascular surface density ($S_v$) by the mean linear intercept method. Vascular length density ($L_v$) was derived from the mean number of vessel segments observed per microscope field (Underwood, 1970), and the standard deviation of these measurements used as an index of vascular heterogeneity ($H_v$). The special eyepiece graticules required for point counting and line intersection counting were obtained from Graticules Ltd, Tonbridge, UK (ref. G52 and GW1).

The relationships used to derive the vascular parameters are summarised in Table I. Between 48 and 80 fields were examined for each region of interest to achieve a relative standard error of approximately 10% for the estimation of $S_v$ and $V_v$. The number of microscope fields necessary to achieve a predetermined level of measurement uncertainty was estimated prior to the study using a set of computer drawn test objects of known circumference and area.

### Results

*Tumour vascularity*

Vascular measurements were successfully completed in a high proportion of the cases (74 normal dermis, 90 tumour and 100 tumour base), the most common reason for failure being the exclusion of fragmented or partly missing tissue sections. In all of but two of the tissue sections UEA-I stained the endothelia of all identifiable vessels, with variable staining of keratin and the epithelial cells associated with hair follicles and sweat glands. Care was taken to avoid the latter areas when morphometric measurements were being made. Examples of the vascular patterns exhibited by the melanomas are shown in Figures 1 and 2. Two distinct patterns were seen in tumour (Figure 1); the vasculature appeared either to be evenly distributed among the tumour cells or was confined to the outer region of tumour cell 'nests' of up to 400 μm diameter. At the tumour base a higher proportion of large calibre vessels was commonly found in the more vascular lesions (Figure 2a), however some lesions were associated with a relatively sparse vasculature in this region (Figure 2b).

The range of values obtained for each of the vascular parameters derived is summarised in Figure 3. A broad distribution of vascularity in the tumour and particularly at the tumour base can be seen relative to that of the adjacent normal dermis. The relatively sparse vascularity found within a proportion of lesions is seen in Figure 3c. In terms of vascular heterogeneity the normal dermis and tumour base were found to be broadly similar with a trend towards greater heterogeneity within the tumour itself. Overall, heterogeneity within tumour was comparable to that between lesions, but at the tumour base greater variation was found between tumours than within individuals.

The wide range of vascularity found at the tumour base compared to normal dermis is illustrated in Figure 4. In the

**Table I** Vascular parameters derived from morphometric measurements

| *Parameter* | *Derivation* | *No. of fields* |
|---|---|---|
| Length density $L_v$ (mm mm$^{-3}$) | $2\,\Sigma\, n/N$ | 20 |
| Surface density $S_v$ (mm$^2$ mm$^{-3}$) | $2\,\Sigma\, n/Nl$ | 60–80 |
| Volume density $V_v$ (%) | $\Sigma\, n/N \times 100$ | 48 |
| Heterogeneity $H_v$ (%) | $\left[\frac{\Sigma n^2-(\Sigma n)^2/N}{N-1}\right]^{\frac{1}{2}} \times \frac{100}{L_v}$ | 20 |

$n$ = number of 'hits' scored by the respective morphometric method. $l$ = 72 μm, the length of test line used for mean linear intercept estimation.

**Figure 1** Vasculature associated with primary cutaneous melanoma, visualised by endothelial staining using *Ulex europaeus* type I lectin showing; **a**, vessels evenly distributed within tumour and **b**, vessels confined to the boundary of tumour cell 'nests'.

**Figure 2** Vasculature in the junctional region between tumour and underlying dermis showing; **a**, dense vascularity including many large calibre vessels and **b**, a tumour with relative sparse vascularity in this region.

normal papillary dermis where vessel calibre is relatively uniform the slope of $S_v$ against $L_v$ gives a diameter of 7.8 µm. This value is within the overall range of 5–10 µm reported for the papillary loops of human skin (Braverman, 1989). The small intercept of the regression line with the ordinate may be explained by the bias introduced in defining the edge of endothelial staining. It may be inferred that the vessels at the tumour base may be of higher or lower 'average' calibre by comparison with the regression line shown.

The relative vascularity of adjacent regions within individual sections is shown in Figure 5. In all cases, vascular surface density at the tumour base was greater or equal to that within tumour, and in general was greater than that of adjacent normal dermis. In contrast, the vascular surface density within tumour was either higher or lower than that of adjacent dermis.

### *Correlation of vascular parameters with clinical and histological features*

The extent of tumour base vascularity in terms of $S_v$ is summarised in relation to those clinical and histological parameters studied in Table III. No explanation could be found in terms of these features for the highly variable degree of vascularity observed.

Of the 107 patients studied 86 remained disease free with a minimum follow-up period of 5 years. Twenty-one patients recurred (Table II). The only clinical feature commonly associated with the recurrence group was the patient's sex. Recurrent disease developed in 9/20 male patients compared with 12/87 females ($\chi^2$ test, $P<0.005$). Using stepwise discriminant analysis, no relationship was found between tumour recurrence and the patient's age, lesion thickness, anatomical site, level of invasion (Clark), degree of lymphocytic infiltration, density of tumour cell mitoses or histological evidence of regression. That tumour vascularity was not related to the likelihood of recurrence is seen in Figure 6. These data are summarised in Table IV along with the previously reported findings of Srivastava *et al.* (1988). Considering the recurrence group alone vascularity was found not to be related to the type of recurrence, disease free interval or duration of patient survival.

### Discussion

The development of a vascular supply is essential for the growth of all solid tumours beyond a size of approximately 1 mm (Folkman, 1985). Vascular access is also an important prerequisite for the metastatic dissemination of tumour cells.

**Figure 3** The distribution of vascularity in cutaneous melanoma and adjacent dermis. ■ – dermis ($n = 74$); ▨ – tumour ($n = 90$); □ – tumour base ($n = 100$).

**Figure 4** The length and surface density of vessels at the tumour base in relation to adjacent dermis. ○ – dermis ($n = 74$); ● – tumour base ($n = 100$). Regression line fitted to dermis data only. ($S_v = 0.0246 \times L_v + 0.770$).

**Figure 5** The relative vascularity of neighbouring regions in primary cutaneous melanoma. ○ – tumour/normal dermis ($n = 68$), Δ – tumour base/normal dermis ($n = 74$), □ – tumour base/tumour ($n = 88$).

This study documents the extent of vasculature associated with cutaneous malignant melanomas of a size consistent with an early stage of this vascular response.

Two potential sources of error must be acknowledged in our quantitative histological method. Firstly it has been assumed that all the vessels identified were functional. It is likely that a proportion of the stained vessels were newly-formed capillary 'sprouts', and therefore would not have contributed to the tumour blood circulation *in situ*. Thus we may have overestimated the effective vascular surface area. Secondly, differences in the ultrastructure of vascular and lymphatic endothelium may influence the relative efficiency of tumour cell intravasation via these routes, and no attempt was made to distiguish vascular from lymphatic vessels in our study. The proportion of lymphatic capillaries in melanoma has been shown to be small (Fallowfield & Cook 1990), and therefore any structural differences were assumed to be of negligible significance.

As a group the lesions studied exhibited a marked degree of vascular heterogeneity, being apparently unrelated to other clinical or histological features. A quantitatively similar distribution of melanoma vascularity was found by Srivastava *et al.* (1986), who showed a significant correlation between vascularity at the lesion base and tumour thickness in the range 0.2–9.0 mm. Our study covered a more restricted thickness range (0.85–1.25 mm), suggesting that if this relationship holds it is non-linear, and is consistent with the findings of a further study by Srivastava *et al.* (1988) of lesions 0.8–3.7 mm in thickness in which no association between tumour thickness and vascularity is apparent. One possibility is that a breakpoint may occur at the clinically significant thickness of 0.76 mm, which may also explain the 'all or nothing' observations of tumour blood flow using Doppler Ultrasound (Srivastava *et al.*, 1986). A larger series of melanomas covering an extended thickness range is needed to verify this.

In this study prognosis was not correlated with tumour vascularity either independently or in combination with other known prognostic variables. These findings do not confirm those of a similar study by Srivastava *et al.* (1988), who showed that tumour vascularity was on average lower in their non-recurrence group. Possibly such a relationship may have been obscured by inflammatory changes or tumour

**Table II** Pattern of initial tumours recurrence

| *Type* | *Incidence* |
|---|---|
| Local | 2 |
| In-transit | 2 |
| Nodal | 7 |
| Systemic | 6 |
| Unspecified | 4 |

**Table III** Tumour base vascularity in relation to clinical and histological features

| *Variable* | *Value* | *Incidence* | *Mean $S_v$ ($mm^2\,mm^{-3}$)* | *± s.d.* |
|---|---|---|---|---|
| *Age (yrs)* | | | | |
| | <35 | 18 | 9.41 | 3.8 |
| | 35–55 | 42 | 9.26 | 5.0 |
| | >55 | 40 | 9.53 | 4.1 |
| *Anatomical site* | | | | |
| | Chest/Abdomen | 5 | 10.9 | 5.6 |
| | Back/Shoulder | 13 | 11.6 | 5.2 |
| | Leg | 58 | 9.41 | 4.4 |
| | Arm | 15 | 8.83 | 3.5 |
| | Head/Neck | 8 | 5.99 | 3.2 |
| *Type of recurrence* | | | | |
| | Local | 2 | 12.0 | – |
| | In-transit | 2 | 12.0 | – |
| | Nodal | 7 | 11.8 | 6.3 |
| | Systemic | 6 | 9.77 | 4.5 |
| *Lesion thickness (mm)* | | | | |
| | <1.00 | 30 | 9.71 | 3.8 |
| | 1.00–1.05 | 27 | 9.04 | 4.5 |
| | >1.05 | 43 | 9.40 | 4.9 |
| *Lymphocytic infiltration* | | | | |
| | None | 3 | 7.26 | – |
| | Minimal | 14 | 8.12 | 3.3 |
| | Mild | 25 | 8.75 | 5.3 |
| | Moderate | 32 | 10.9 | 4.5 |
| | Maximal | 26 | 9.12 | 4.0 |
| *Regression* | | | | |
| | None | 29 | 7.73 | 3.6 |
| | Minimal | 6 | 5.41 | 2.1 |
| | Mild | 21 | 9.63 | 5.0 |
| | Moderate | 20 | 11.2 | 4.2 |
| | Maximal | 17 | 11.1 | 4.4 |
| *Frequency of mitoses* | | | | |
| | Low (< 1 per field) | 41 | 10.2 | 4.1 |
| | High (⩾ 1 per field) | 54 | 8.74 | 4.8 |

**Figure 6** Clinical outcome in relation to tumour vascularity within **a**, the tumour base region (○ – non-recurrence, $n = 77$; ● – recurrence, $n = 20$) and **b**, the tumour (○ – non-recurrence, $n = 70$; ● – recurrence, $n = 17$).

**Table IV** Tumour vascularity and prognosis

| | *Parameter* | *Disease free ± s.d. (no. of cases)* | *Recurrence ± s.d. (no. of cases)* |
|---|---|---|---|
| *Tumour* | $S_v$(mm² mm⁻³) | 5.04 ± 2.8 ($n = 70$) | 4.66 ± 3.0 ($n = 17$) |
| | $V_v$ (%) | 2.45 ± 2.0 ($n = 70$) | 2.36 ± 2.0 ($n = 17$) |
| *Tumour base* | $S_v$(mm² mm⁻³) | 9.18 ± 4.5 ($n = 77$) | 10.2 ± 4.5 ($n = 20$) |
| | $V_v$ (%) | 5.76 ± 4.5 ($n = 77$) | 6.89 ± 5.0 ($n = 20$) |
| | $V_v$ (%)[a] | 1.57 ± 1.3 ($n = 10$) | 4.60 ± 3.3 ($n = 10$) |

[a]Data from Srivastava *et al.*, 1988.

regression at the time of surgery, but this is not supported by our histological findings. Further, the incidence of spontaneous regression in primary cutaneous melanoma is commonly reported to be low (McGovern & Murad, 1985).

The variable extent of tumour vascularity, if expressed by melanoma metastases also, could have implications for the local distribution of pharmaceuticals intended for diagnosis or therapy. If the surface area of the vascular bed is found to be consistently higher at the tumour periphery compared with adjacent tissue, then the detection of occult lesions on this basis is worthy of investigation. Considering cytotoxic drug delivery however, penetration beyond the peripheral cells into the tumour may be expected to be highly non-uniform for compounds unable to diffuse freely within the interstitium. The presence of large calibre vessels within the region of the tumour base suggests that favourable manipulation of drug delivery using intravascular microsphere blockage may be feasible.

Our findings suggest that vascular morphometry as applied to cutaneous melanoma is not helpful prognostically within the range of tumour thickness chosen. In this respect, the assessment of other features of the tumour vasculature may provide a useful complement to simple morphometry.

The authors gratefully acknowledge the financial support of the Cancer Research Campaign UK, and the MN Foundation.

### References

BRAVERMAN, I.M. (1989). Ultrastructure and organization of the cutaneous microvasculature in normal and pathologic states. *J. Invest. Dermatol.*, **93**, 2S.

BRESLOW, A. (1970). Thickness, cross-sectional areas and depth of invasion in the prognosis of cutaneous melanoma. *Ann. Surg.*, **172**, 902.

BRIGGS, J.C. (1985). Cutaneous melanoma: the use of a computer to aid in the investigation of the disease. *Pathology*, **17**, 328.

CHALKLEY, H.W. (1943). Method for the quantitative morphologic analysis of tissues. *J. Natl Cancer Inst.*, **4**, 47.

DAY, C.L., MIHM, M.C., SOBER, A.J. & 18 others (1982). Prognostic factors for melanoma patients with lesions 0.76–1.69 mm in thickness. *Ann. Surg.*, **195**, 30.

DAY, C.L., MIHM, M.C., LEW, R.A. & 18 others (1982). Prognostic factors for patients with clinical stage I melanoma of intermediate thickness (1.51–3.99 mm). *Ann. Surg.*, **195**, 35.

FALLOWFIELD, M.E. & COOK, M.G. (1990). Lymphatics in primary cutaneous melanoma. *Am. J. Surg. Pathol.*, **14**, 370.

FOLKMAN, J. (1985). Tumour angiogenesis. *Adv. Cancer Res.*, **43**, 175.

HOLTHÖFER, H., VIRTANEN, I., KARINIEMI, A.-L., HORMIA, M., LINDER, E. & MIETTINEN, A. (1982). *Ulex europaeus* I Lectin as a marker for vascular endothelium in human tissues. *Lab. Invest.*, **47**, 60.

MCGOVERN, V.J. & MURAD, T.M. (1985). Pathology of melanoma: an overview. In *Cutaneous Melanoma. Clinical Management and Treatment Results Worldwide*. Balch, C.M. & Milton, G.W. (eds), p. 29. Lippincott: Philadelphia.

SRIVASTAVA, A., LAIDLER, P., HUGHES, L.E., WOODCOCK, J. & SHEDDEN, E.J. (1986). Neovascularization in human cutaneous melanoma: a quantitative morphological and Doppler ultrasound study. *Eur. J. Cancer Clin. Oncol.*, **22**, 1205.

SRIVASTAVA, A., LAIDLER, P., DAVIES, R.P., HORGAN, K. & HUGHES, L.E. (1988). The prognostic significance of tumour vascularity in intermediate-thickness (0.76–4.0 mm thick) skin melanoma. *Am. J. Pathol.*, **133**, 419.

SRIVASTAVA, A., HUGHES, L.E., WOODCOCK, J.P. & LAIDLER, P. (1989). Vascularity in cutaneous melanoma detected by Doppler sonography and histology: correlation with tumour behaviour. *Br. J. Cancer*, **59**, 89.

UNDERWOOD, E.E. (1970). *Quantitative Stereology*. Addison-Wesley: Reading, Mass.

WEIBEL, E.R. (1963). Principles and methods for the morphometric study of the lung and other organs. *Lab. Invest.*, **12**, 131.